# Bacterial and Archaeal Viruses of Himalayan Hot Springs at Manikaran Modulate Host Genomes

**DOI:** 10.3389/fmicb.2018.03095

**Published:** 2018-12-14

**Authors:** Anukriti Sharma, Matthias Schmidt, Bärbel Kiesel, Nitish K. Mahato, Lauren Cralle, Yogendra Singh, Hans H. Richnow, Jack A. Gilbert, Wyatt Arnold, Rup Lal

**Affiliations:** ^1^Department of Zoology, University of Delhi, New Delhi, India; ^2^Biosciences Division, Argonne National Laboratory, Lemont, IL, United States; ^3^Department of Surgery, University of Chicago, Chicago, IL, United States; ^4^Department of Isotope Biogeochemistry, Helmholtz Centre for Environmental Research, Leipzig, Germany; ^5^Department of Environmental Microbiology, Helmholtz Centre for Environmental Research, Leipzig, Germany

**Keywords:** archaeal viruses, bacteriophages, scanning-electron microscopy, helium-ion microscopy, Caudovirales

## Abstract

Hot spring-associated viruses, particularly the archaeal viruses, remain under-examined compared to bacteriophages. Previous metagenomic studies of the Manikaran hot springs in India suggested an abundance of viral DNA, which prompted us to examine the virus–host (bacterial and archaeal) interactions in sediment and microbial mat samples collected from the thermal discharges. Here, we characterize the viruses (both bacterial and archaeal) from this Himalayan hot spring using both metagenomics assembly and electron microscopy. We utilized four shotgun samples from sediment (78–98°C) and two from microbial mats (50°C) to reconstruct 65 bacteriophage genomes (24–200 kb). We also identified 59 archaeal viruses that were notably abundant across the sediment samples. Whole-genome analyses of the reconstructed bacteriophage genomes revealed greater genomic conservation in sediments (65%) compared to microbial mats (49%). However, a minimal phage genome was still maintained across both sediment and microbial mats suggesting a common origin. To complement the metagenomic data, scanning-electron and helium-ion microscopy were used to reveal diverse morphotypes of Caudovirales and archaeal viruses. The genome level annotations provide further evidence for gene-level exchange between virus and host in these hot springs, and augments our knowledgebase for bacteriophages, archaeal viruses and Clustered Regularly Interspaced Short Palindromic Repeat cassettes, which provide a critical resource for studying viromes in extreme natural environments.

## Introduction

Viruses are the most prominent predators and mediators of genetic transmission in prokaryotic communities in extreme thermal environments, which are characterized by lack of eukaryotes (Breitbart et al., 2004). The majority of well-characterized prokaryotic viruses belong to the order Caudovirales, which are bacteriophages with tails (Krupovic et al., 2011). In the past, the sequence-based viral characterization as well as electron and ion microscopy have been used for elucidating viral diversity within geothermally heated ecosystems (Häring et al., 2005). A series of Himalayan hot springs (surface temperature > 95°C) located at an altitude of 1,760 m at Manikaran (32°02′N, 72°21′E) have previously been investigated using sequencing approaches to characterize the bacterial communities (Dwivedi et al., 2012; Mahato et al., 2014; Sharma et al., 2014, 2016b; Tripathi et al., 2016). Culture-independent analysis of the sediment and microbial mat samples demonstrated unexpectedly high microbial diversity in these hot springs (Sangwan et al., 2015; Sharma et al., 2016a). Sediment samples with temperatures ranging from 78 to 98°C were dominated by archaeal genotypes, while microbial mats maintained high abundance of resident bacterial populations with integrated phage DNA (Sangwan et al., 2015). However, the role of these viruses in microbial community dynamics remains undetermined.

We explored the virome of these Himalayan hot springs by coupling metagenomic profiling of the bacteriophages and archaeal viruses with *in situ* electron microscopy of virus-enriched sediment and microbial mat samples. We assembled four metagenomes from sediments at a range of temperatures [98°C (MnS1), 85°C (MnS2), 81°C (MnS3), 85°C (MnS4)], and two from microbial mat samples (MM1, MM2; 50°C), in order to reconstruct 65 phage genomes. Moreover, 59 different archaeal viruses were found to be abundant in the sediment samples. We also reconstructed Clustered Regularly Interspaced Short Palindromic Repeats (CRISPR) cassettes, and the genomes of potential bacterial hosts. This allowed us to (1) determine and characterize the potential phage ensemble at the Manikaran hot springs, and (2) explore the gene contribution of phages to their bacterial and archaeal hosts. Furthermore, the use of electron microscopy on sediment and mat samples allowed us to visualize and make cladistics assignments to the prokaryotic virosphere especially the archaeal viruses since the archaeal reference sequence databases still remain limited (Häring et al., 2005).

## Results and Discussion

### Viral Diversity in Microbial Mat and Sediment Samples

The Himalayan hot springs located at Manikaran were analyzed for prokaryotic viral genomic signatures. These hot springs are characterized by abundance of Fe (12,380 ug g^-1^), Mn (2,189 ug g^-1^), As (80 ug g^-1^) and Se (1.5 ug g^-1^). Previously we demonstrated that the microbial mats at Manikaran are enriched in bacterial genera such as *Enterobacter, Bdellovibrio, Clostridium*, and *Achromobacter* (Sangwan et al., 2015). Furthermore, we reconstructed the genome of a novel *Enterobacter* strain with its corresponding bacteriophage (Sangwan et al., 2015), suggesting a potential phage–host dynamics at the Manikaran hot springs, which became the focus of the current study.

Metagenomic data from the microbial mat (*n* = 2; 50°C) and sediment samples (*n* = 4, 78–98°C) (Table 1 and Supplementary Figure S1) was assembled for binning phage genomes. We expected differential viral enrichments in the moderately mesophilic microbial mats compared to the relatively thermophilic sediment samples (78–98°C), which were dominated by Archaea (Sangwan et al., 2015). While water samples (95°C) were collected, we were unable to detect sufficient phage DNA (based on BLAST results against viral database) in the metagenomic sequence data, and thus water samples were excluded from the analyses.

**Table 1:** Characteristics of the metagenome samples analyzed from the Manikaran hot springs.

S. No	Site	Assembly size (Mb)	Temp. (°C)	pH	Bacteriophages reconstructed	Size of the largest bacteriophage (kb)	CRISPR cassettes	Number of archaeal viral contigs
1	MM1	95	50	7	5	200	45	70
2	MM2	13	50	7	9	104.9	7	52
3	MnS1	64	98	7.3	28	42.2	307	2713
4	MnS2	53	85	7.1	6	24.3	54	1400
5	MnS3	18	81	7.1	7	37.5	17	1180
6	MnS4	74	78	7.6	11	25.3	77	5132

**FIGURE 1:**
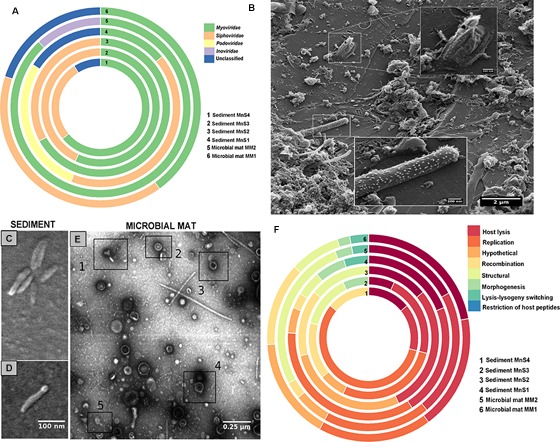
**(A)** Stack graph showing relative abundance (%) of Caudovirales phages reconstructed across sediment (MnS1, MnS2, MnS3, MnS4) and microbial mat samples (MM1 and MM2). **(B)** The scanning helium-ion micrographs display typical areas of the microbial bio-films found in the sediment. Sediment is microbial deposition found around the thermal discharges at depth of 10 cm from the surface where the hot spring water flows out. These samples show that phage/virus infected bacteria were found. The insets in **(B)** show the viral particles as well a dead host cell in the vicinity. **(C,D)** Scanning electron micrographs of viruses enriched from sediment samples showing spindle shaped archaeal viruses with identical scale bar. **(E)** Scanning transmission electron micrograph of phages enriched from the mat samples showing different morphotypes: 1. *Myoviridae*, 2. Tailless phage, 3. Tailless phage head, 4. *Siphoviridae*, 5. *Podoviridae*. All the images were collected at electron energy of 10 kV and a beam current of 250 pA. **(F)** Stack graphs showing relative abundance of eight categories of functional genes annotated from Caudovirales phages reconstructed across sediment (MnS1, MnS2, MnS3, MnS4) and microbial mat samples (MM1 and MM2). The eight broad functional categories were formed from 99 orthologous genes identified in the reconstructed genomes using the Hidden Markov Model (HMM) profiles for orthologous genes from Caudovirales.

In total, 709 contigs were identified as having sequence similarity to bacteriophage, 103 of which were found in microbial mat samples, while 606 originated in the sediment samples (Table 1). The contigs were then re-assembled and curated into 65 bacteriophage genomes (Table 2), of which 90% were assigned to the order Caudovirales. Caudovirales are the most abundant and well characterized viruses (Krupovic et al., 2011), as a result, extensive reference databases exist for the analysis and annotation (Munson-McGee et al., 2018). The majority (71%; 10/14) of bacteriophage genomes from microbial mat samples belonged to the family *Myoviridae*, whereas genomes reconstructed from the sediment samples were assigned either to *Myoviridae* (39%; 20/51), *Siphoviridae* (35%; 18/51), or *Podoviridae* (16%; 8/51) (Figure 1A and Table 2). *Siphoviridae* were most abundant in the high temperatures (sediments), while *Myoviridae* phages were most populous at samples with lower temperatures (microbial mats) (Table 2).

**Table 2:** Characteristics of phages reconstructed across microbial mat and sediment of Manikaran hot springs.

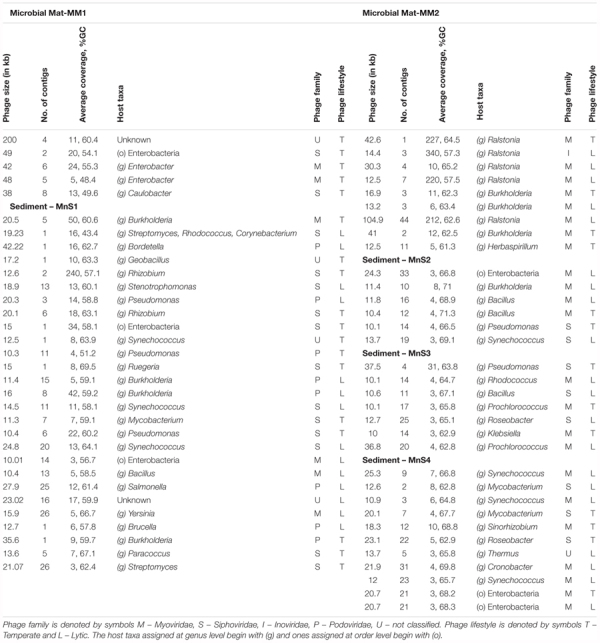

Using a database of 95 archaeal virus genomes from NCBI, we were able to identify metagenomic contigs associated with 59 different archaeal viruses, which originated primarily from the sediment samples (Table 3 and Supplementary Figure S2), corresponding to the dominance of Archaea in this environment. At the family level, these contigs were assigned to the archaeal virus families of *Fuselloviridae, Siphoviridae*, and *Lipothrixviridae* (Table 3 and Supplementary Figure S2). The detailed annotations of these archaeal virus contigs are mentioned in Table 3. This is only the second study to report a relatively novel archaeal turreted icosahedral virus with *Metallosphaera* (MT1V1 strain) as the archaeal host, which was recently isolated from Yellowstone National Park (YNP) (Wagner et al., 2017) (Table 3). While, we were able to reconstruct draft genomes for bacteriophages, we were not able to successfully assemble draft archaeal virus genomes potentially due to low sequencing depth and lack of biological replicates (Albertsen et al., 2013; Nielsen et al., 2014; Sangwan et al., 2016).

**Table 3:** The BLASTX results of metagenome contigs from sediments and mat samples, searched against the archaeal virus sequence database.

S. No	Best blast hit (BBH) annotation of contigs	Archaeal virus family	MNS1 (# of BBH, average %identity)	MNS2 (# of BBH, average %identity)	MNS3 (# of BBH, average %identity)	MNS4 (# of BBH, average %identity)	Mat (# of BBH, average %identity)
1	*Halovirus* HHTV-1	*Fuselloviridae*	31, 90	25, 89	13, 91	53, 88	7,90
2	*Acidianus* bottle-shaped virus 2 strain ABV2	*Lipothrixviridae*	73, 55	31, 60	18, 51	110, 57	3, 61
3	*Acidianus* bottle-shaped virus 3 strain ABV3	*Lipothrixviridae*	92, 57	30, 54	26, 60	165, 56	3, 57
4	*Acidianus* filamentous virus 1	*Lipothrixviridae*	51, 60	11, 61	27, 59	71, 58	1, 60
5	*Acidianus* filamentous virus 2	*Lipothrixviridae*	78, 52	37, 54	28, 58	151, 55	2, 51
6	*Acidianus* filamentous virus 3, partial viral genome	*Lipothrixviridae*	0	24, 60	12, 56	119, 61	2, 59
7	*Acidianus* filamentous virus 6	*Lipothrixviridae*	72, 62	34, 61	13, 63	112, 64	4, 61
8	*Acidianus* filamentous virus 7	*Lipothrixviridae*	68, 70	31, 72	13, 71	119, 70	9, 71
9	*Acidianus* rod-shaped virus 1	*Lipothrixviridae*	53, 52	23, 51	14, 50	97, 53	7, 54
10	*Acidianus* rod-shaped virus 2 strain ARV2	*Lipothrixviridae*	94, 51	53, 56	48, 49	154, 42	7, 56
11	*Acidianus* spindle-shaped virus 1	*Lipothrixviridae*	46, 59	20, 53	12, 51	92, 67	3, 53
12	*Acidianus* two-tailed virus	*Lipothrixviridae*	94, 51	47, 62	70, 63	145, 53	4, 61
13	*Aeropyrum pernix ovoid* virus 1 complete genome	*Clavaviridae*	0	3, 58	0	17, 62	4, 67
14	*Aeropyrum pernix* spindle-shaped virus 1 complete genome	*Clavaviridae*	0	12, 55	0	43, 58	1, 49
15	Archaeal BJ1 virus	*Siphoviridae*	23, 58	5, 61	3, 67	35, 68	3, 63
16	*Haloarcula hispanica* icosahedral virus 2	*Fuselloviridae*	17, 59	8, 63	10, 62	21, 61	6, 55
17	*Haloarcula hispanica* pleomorphic virus 1	*Fuselloviridae*	3, 67	3, 55	5, 63	8, 61	8, 50
18	*Haloarcula hispanica* pleomorphic virus 2	*Fuselloviridae*	3, 59	1, 62	2, 57	15, 67	2, 43
19	*Haloarcula* phage SH1	*Fuselloviridae*	16, 67	4, 51	16, 50	23, 53	5, 60
20	*Halogeometricum* pleomorphic virus 1	*Pleolipoviridae*	4, 56	2, 58	1, 55	6, 50	1, 59
21	*Halorubrum* phage HF2	*Pleolipoviridae*	11, 55	8, 56	7, 67	32, 60	5, 50
22	*Halorubrum* pleomorphic virus 1	*Pleolipoviridae*	9, 52	4, 54	9, 53	7, 68	6, 55
23	*Halorubrum* pleomorphic virus 3	*Pleolipoviridae*	5, 67	5, 56	5, 50	14, 51	1, 56
24	*Halovirus* HCTV-1	*Fuselloviridae*	62, 59	29, 58	9, 54	99, 51	5, 57
25	*Halovirus* HCTV-2	*Fuselloviridae*	38, 57	16, 67	20, 61	39, 63	2, 67
26	*Halovirus* HF1	*Fuselloviridae*	45, 68	22, 69	13, 73	73, 60	4, 72
27	*Halovirus* HGTV-1	*Fuselloviridae*	148, 67	64, 53	60, 54	235, 71	1, 61
28	*Halovirus* HRTV-4	*Fuselloviridae*	32, 54	12, 59	10, 54	37, 67	1, 52
29	*Halovirus* HRTV-8	*Fuselloviridae*	65, 61	13, 64	12, 60	66, 55	3, 51
30	*Halovirus* HSTV-1	*Fuselloviridae*	26, 57	8, 56	18, 60	25, 59	6, 45
31	*Halovirus* HSTV-2	*Fuselloviridae*	30, 43	10, 58	23, 60	76, 61	4, 63
32	*Halovirus* HVTV-1	*Fuselloviridae*	55, 53	24, 57	18, 51	89, 60	9, 52
33	*Halovirus* PH1	*Fuselloviridae*	19, 60	1, 55	0	23, 61	1, 62
34	*Halovirus* VNH-1 genomic sequence	*Fuselloviridae*	10, 59	2, 56	1, 61	20, 65	7, 56
35	His1 virus	*Fuselloviridae*	28, 56	15, 61	1, 55	39, 54	9, 60
36	Hyperthermophilic archaeal virus 1	Unknown	28, 55	13, 40	2, 51	50, 53	7, 42
37	Hyperthermophilic archaeal virus 2	Unknown	13, 49	4, 50	5, 55	24, 60	8, 54
38	*Metallosphaera* turreted icosahedral virus strain MTIV1	Unknown	4, 59	2, 60	1, 61	15, 64	3, 65
39	*Methanobacterium* phage psiM2	*Siphoviridae*	30, 61	13, 56	10, 59	46, 62	3, 56
40	*Natrialba* phage PhiCh1	*Myoviridae*	30, 60	18, 68	18, 70	53, 63	8, 51
41	*Pyrobaculum* filamentous virus 1	*Globuloviridae*	32, 59	13, 61	13, 63	47, 76	6, 44
42	*Pyrobaculum* spherical virus	*Globuloviridae*	22, 55	15, 51	12, 49	53, 61	8, 45
43	*Pyrococcus abyssi* virus 1	*Rudiviridae*	24, 60	11, 55	10, 61	28, 59	8, 63
44	*Sulfolobales* Mexican fusellovirus 1	*Fuselloviridae*	19, 56	6, 67	3, 55	33, 49	9, 45
45	*Sulfolobales* Mexican rudivirus 1	*Fuselloviridae*	28, 71	18, 60	13, 63	61, 67	4, 51
46	*Sulfolobales* virus YNP1 strain SYV1	*Fuselloviridae*	70, 52	29, 67	40, 54	137, 61	8, 50
47	*Sulfolobales* Virus YNP2 strain SYV2	*Fuselloviridae*	54, 58	14, 60	15, 67	75, 66	9, 79
48	*Sulfolobus islandicus* filamentous virus	*Rudiviridae*	111, 55	47, 61	89, 51	197, 59	4, 41
49	*Sulfolobus islandicus* rod-shaped virus 10, partial genome	*Rudiviridae*	0	101, 67	10, 51	351, 49	6, 60
50	*Sulfolobus islandicus* rod-shaped virus 2	*Rudiviridae*	219, 59	84, 60	78, 55	324, 63	5, 61
51	*Sulfolobus islandicus* rudivirus 3 isolate SIRV3	*Rudiviridae*	196, 55	116, 58	120, 60	313, 61	5, 59
52	*Sulfolobus monocaudavirus* SMV1 complete genome	*Rudiviridae*	0	40, 61	28, 59	113, 64	3, 61
53	*Sulfolobus monocaudavirus* SMV4	*Rudiviridae*	82, 55	38, 49	18, 52	144, 51	1, 56
54	*Sulfolobus* turreted icosahedral virus	*Rudiviridae*	49, 55	21, 56	19, 54	75, 55	7, 54
55	*Sulfolobus* virus 1 complete genome (provirus)	*Rudiviridae*	0	20, 67	19, 71	56, 55	9, 49
56	*Sulfolobus* virus Kamchatka 1	*Rudiviridae*	38, 61	15, 65	20, 68	56, 62	0
57	*Sulfolobus* virus Ragged Hills	*Rudiviridae*	38, 61	21, 71	12, 73	55, 60	0
58	*Sulfolobus* virus STSV2	*Rudiviridae*	198, 59	96, 58	70, 55	348, 57	3, 58
59	*Thermococcus prieurii* virus 1	*Fuselloviridae*	27, 50	8, 49	18, 55	48, 56	8, 59

*The BLAST output was parsed for Best Blast Hits (BBH) based on E-value, bit score, and coverage cut-off of 80%. The BBH annotations for the metagenome contigs that showed a match are mentioned in the table along with % identity (average) and number of BBH hits for each archael virus type (i.e., number of contigs showing a match to the archaeal virus).*

Electron microscopy and helium-ion microscopy (HIM) was used to visualize viruses in these samples. HIM revealed virus-like particles of 50–90 nm attached to the surface of bacteria (Figure 1B). HIM was able to capture good resolution images even though we used heterogeneous mat and sediment samples directly. These images paralleled what was seen in electron micrographs obtained from virus–host infections in pure-cultures (Rice et al., 2001; Rachel et al., 2002; Häring et al., 2005). These results led us to further explore pure virus enrichments from sediment and microbial mat samples, which could then be examined even under the scanning electron microscope (SEM) at a high enough resolution to refine the taxonomic assignments.

The spindle-shaped viruses were found in the sediment samples, which were characterized by diverse range of sizes. These were then assigned to the family *Fuselloviridae* based on their morphology (Figures 1C,D) (Prangishvili, 2013). This family was found to be abundant across sediment samples based on metagenomic DNA as well. However, we were not able to visualize other families such as *Siphoviridae* and *Lipthrixviridae* (identified using sequence-based analyses) suggesting that our current culturing methodology was not able to enrich these viral families. The smallest archaeal viral particle observed via electron microscopy was 17 nm in width and 60 nm in length, while the largest was 40 nm in width and 200 nm in length (data not shown). Interestingly, a high resolution magnified image of one of these viral particles revealed two different asymmetric ends, which is consistent with reports indicating that archaeal viruses belonging to family *Fuselloviridae* have a ‘mouth’ and ‘tail’ (host attachment and penetration is achieved through the ‘mouth’) (Figure 1D) (Prangishvili et al., 2013). The electron microscopy of the four sediment samples revealed an abundance of archaeal viruses of a similar spindle-shaped morphotype. We further visualized all sediment enrichments and the same spindle shaped *Fuselloviridae* morphotype was always present (two examples are shown in Figure 1D).

The microbial mat samples were characterized by a wide variety of bacteriophages belonging to different tailed (*Myoviridae, Siphoviridae, Podoviridae*) and tailless (single and double membrane bound viral particles) families (Figure 1E) (Clokie et al., 2011). These different families each have their own characteristic morphology, with *Myoviridae* particles having a head size of 50–110 nm (Figure 1E), *Siphoviridae* phages having a head size of 50–70 nm (Figure 1E), and *Podoviridae* phages possessing a very small tail that is difficult to observe via scanning electron microscopy (Clokie et al., 2011) (though we were still able to image a *Podoviridae* particle) (Figure 1E, element 5). This diversity of bacteriophages within microbial mats is perhaps best attributed to the moderately mesophilic (up to ∼50°C) environments, that allow for more bacterial host diversity (Pietilä et al., 2014). Archaea are better at surviving the extreme temperatures found in the sediment samples, and so outcompete bacteria (Munson-McGee et al., 2018), resulting in an increased abundance of archaeal viruses.

### Phylogenomic Clustering Analysis

As phage genomes lack a credible set of marker genes, we relied on the total predicted proteome (based on the complete genome sequence data of each phage) for phylogenomic delineation (Rohwer and Edwards, 2002). Sediment samples showed overall site-specific clustering, whereas microbial mat phages were distributed throughout the phylogeny (Supplementary Figure S3), which is supported by the greater genomic conservancy in the sediment samples. Among the sediment samples, phage genomes associated with the MnS1 clustered together. The phage genomes recovered from microbial mats were not seen to cluster together in the proteome-based phylogenetic tree, presumably due to the relatively low number of phage (nodes) representatives from mats in comparison to the sediment samples. Interestingly, the family level assignments for both microbial mat and sediment phage genomes were not tight clades, but rather were distributed throughout the entirety of the phylogenetic tree (Supplementary Figure S3). However, small sub-clades were observed for the family *Myoviridae* (Supplementary Figure S3). This could potentially be due to a greater diversity of phage genomes, as well as a result of the lack of a sound methodology for reconstructing phage phylogenies (Hatfull et al., 2010). Additionally, this could be attributed to incomplete databases, as only a small number of phage genomes have complete family level characterization, or to the potential novelty of the phage data analyzed in this study.

### Functional Characterization of Phage Genomes

Functional annotation was used to predict the lifestyles of the assembled bacteriophage genomes using the Phage Classification Tool Set (PHACTS) (McNair et al., 2012). The proteins involved in integration, excision, lysogeny, regulation of expression and toxins genes are predominantly important toward classifying temperate phages, whereas proteins involved in nucleotide metabolism, phage lysis and structural Proteins are predominantly important toward classifying virulent phages (McNair et al., 2012). Using supervised learning based Random Forest classifier PHACTS can predict the phage genome lifestyle with an average accuracy of 99%. The reconstructed MM1 phages were predicted to have temperate (44%) and lytic (56%) lifestyles (Table 2). However, all the reconstructed MM2 phages were predicted to be temperate in lifestyle (100%). Meanwhile, sediment phages were predicted to be both lytic (MnS1 = 50%, MnS2 = 67%, MnS3 = 57%, and MnS4 = 63%) and temperate (MnS1 = 50%, MnS2 = 33%, MnS3 = 43%, and MnS4 = 37%) (Table 2). These results are, however, based on the reconstructed phage genomes only and thus cannot represent the lifestyle of total bacteriophage community in these samples.

Hidden Markov Model (HMM) profiles for orthologous genes from *Caudovirale*s were used to assign function to the genes in the assembled genotypes. In total, 99 orthologous genes were annotated and further clustered into eight functional categories: host lysis, replication, hypothetical proteins, recombination, structural, morphogenesis, lysis-lysogeny switching, and restriction of host peptides (Supplementary Table S1 and Figure 1F). All the phage genomes presented hypothetical proteins (27% ± 12), as well as genes involved with replication (21% ± 4), host lysis (15% ± 4), morphogenesis (12% ± 8), recombination (11% ± 9), and structure (10% ± 5) (Figure 1F).

### Comparative Genomics of Reconstructed Phage Genomes

**FIGURE 2:**
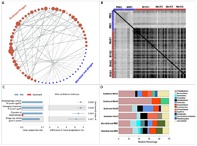
**(A)** Network analysis performed between different phage genomes (reconstructed from both microbial mats and sediments based on tetranucleotide frequencies using average distance matrix. The networks were prepared in igraph. The nodes here stand for the different phage genomes; the node size is controlled by weight of the edges connecting the node. The edges of the network stand for correlation between the phage genomes based on abundance of tetranucleotide frequencies. The weights of the edges are based on the correlation values based on tetranucleotide frequencies between nodes. **(B)** Dot plot analysis of 65 tailed bacteriophage reconstructed across MM1, MM2, sediment samples (MnS1, MnS2, MnS3, and MnS4) based on whole-genome alignment score generated by Kalign. The dot plots were produced in Gepard at a word size setting of 10. **(C)** Differentially abundant (*p* < 0.05, Welch’s two-group *t*-test) functional gene orthologs between phage genomes from sediment and microbial mats, and **(D)** phylum level assignment of CRISPR arrays across Manikaran metagenomes, i.e., microbial mat (MM1 and MM2) and sediments (MnS1, MnS2, MnS3, MnS4).

The phage genomes (*n* = 65) were compared based on whole genome alignments using dot plots and network plots based on tetranucleotide frequencies. Low sequence similarity was observed between the phages assembled from the microbial mats (49 ± 1.6%) and high sequence similarity (65 ± 1%) was observed for those assembled from the sediment (Figures 2A,B). Additionally, we identified differentially abundant (*p* < 0.05, Welch’s *t*-test) functional gene orthologs between phage genomes from microbial mats and sediment samples (Figure 2C). The structural phage proteins including packaging chaperon FI (gpFI) and FII (gpFII) proteins, phage late control gene D protein, and CI repressor protein (Figure 2C) were higher (*p* < 0.05) in abundance in microbial mats. CI repressor proteins play significant role in phages by determining the lifestyle, i.e., lytic or lysogenic (Pedersen et al., 2010).

A set of shared orthologs were found between sediment (*n* = 4) and microbial mat (*n* = 2) phage genomes, including genes coding for structural phage proteins, such as phage portal protein, integrase, baseplate assembly proteins, and phage related replication machinery. Notably, the phage genomes reconstructed from both sediment and microbial mat samples also carried a common core encoding for non-phage proteins, such as hybrid sensor histidine kinase, an arsenic resistance operon repressor, Clp protease, hydroxypyruvate reductase, and methyltransferase. The presence of integrase genes across both sample types suggests that lysogeny is common in thermal discharges, which is consistent with previous studies (Schoenfeld et al., 2008). Additionally, the bacterial genes encoding Clp protease, DNA methyltransferases, and histidine kinase in the phage genomes have been implicated in regulation of protein synthesis, removal of misfolded proteins, antibiotic resistance and virulence in bacteria (Krüger et al., 2000; Pedersen et al., 2010; Tang et al., 2013; Malik and Brötz-Oesterhelt, 2017). These results suggest a role for viral transmission of these functions in bacterial fitness via lateral gene transfers.

Site-specific phage orthologous proteins were identified in all sediment (MnS1–MnS4) and microbial mat samples (MM1, MM2) so that the influence of micro-niches on the genetic repertoire of the phage community could be determined. We again identified presence of both phage and non-phage proteins among the set of shared orthologous proteins at both sites. The orthologous proteins (*n* = 28) between the genomes of phages reconstructed from microbial mat samples, included transporter units, SbcC phage protein, and RnhA endonuclease protein. The reconstructed sediment phages also shared common orthologs (*n* = 49) including both phage and non-phage genes. Besides the phage-related replicative and structural machinery, the genomes contained regions coding for TonB-dependent receptors, histidine kinase, ClpC protease, thymidylate synthase, transposase, integrase, etc. TonB-dependent receptors play a role in infection for *Siphoviridae* phages (Rabsch et al., 2007). Histidine kinase was recently reported in phage genomes for the first time and implicated directly in bacterial biofilm formation and also pathogenicity (Hargreaves et al., 2014). The presence of these genes, along with phage-related genes, integrases, and transposases, among orthologs suggest potential lateral gene transfer activity between these phage genomes and their hosts in this extreme environment.

### Metagenomic Reconstruction and Motif Level Assignment of CRISPR Cassettes

Since viruses are predatory in nature, it is unsurprising that both bacterial and archaeal hosts have developed a countermeasure—the CRISPR system—which confers adaptive immunity against future viral attacks (Rath et al., 2015). The presence of intact CRISPR arrays provide molecular evidence of prior viral infections and resulting adaptive immunity. CRISPR cassettes were predicted using both PILER-CR and CRISPRFinder, with the consistent arrays retained in the hopes of minimizing the false positives. The final set of CRISPRs consisted of 7, 45, and 455 cassettes from MM2, MM1, and sediment (MnS1 = 307, MnS2 = 54, MnS3 = 17, MnS4 = 77), respectively. CRISPR cassettes were found to be composed of copies of a ∼35-nucleotide (nt) direct repeat (DR) region separated by ∼34-nt long spacer sequences. Each repeat sequence of a CRISPR cassette is known to be unique and thus is used to characterize the CRISPR type (Skennerton et al., 2013). Among the total set of 507 CRISPR arrays, 4, 23, 14, 19, 6, and 21 different CRISPR cassettes with unique repeat type were found across MM2, MM1, and sediments – MnS1, MnS2, MnS3, and MnS4, respectively (see Supplementary Table S2). Out of 87 distinct CRISPR types, the majority (35.6%) was assigned to phylum Proteobacteria, followed by Actinobacteria (8%), Firmicutes (8%), Euryarchaeota (8%), and Bacteroidetes (6.9%) (Figure 2D). Five CRISPR cassettes were related to Crenarchaeota (5.7%) and three each from Cyanobacteria and Chloroflexi (Figure 2D). Only two CRISPR cassettes were assigned a species level designation of *Oscillochloris trichoides* (Figure 2D). This analysis also suggests that there is an uneven distribution of CRISPRs across the prokaryotic phyla. Recent explanations of this phenomena point to the predominance of symbiotic lifestyles in bacteria, or the potential that the maintenance costs a CRISPR systems outweigh the conferred defensive benefits due to frequent auto-immunity (Burstein et al., 2016).

**FIGURE 3:**
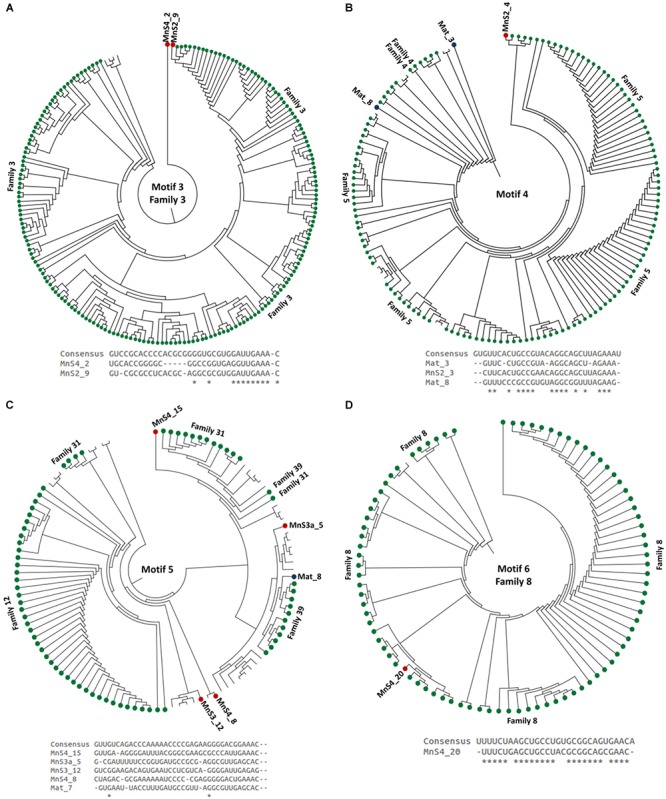
Dendrograms showing the relationship between repeat consensus sequences of the CRISPR cassettes belonging to **(A)** motif 1, **(B)** Motif 2, **(C)** Motif 3, and **(D)** Motif 4, identified across microbial mat and sediment metagenomes. The red and blue nodes represent the repeat sequences belonging to the sediment and microbial mats. Green nodes represent the sequences in the CRISPR database with the family level assignments. All the sequences here were used from the CRISPR database with known motif level assignments. Below each dendrogram, there is an alignment between the consensus repeat sequences generated for a specific CRISPRs motif and the repeat sequences identified in microbial mat and sediment samples.

**FIGURE 4:**
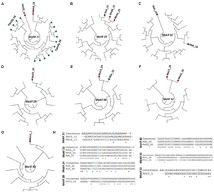
**(A–G)** Dendrogram showing the relationship between repeat consensus sequences of motifs of CRISPR cassettes identified across microbial mat and sediment metagenomes, where red and blue nodes represent the repeat sequences belonging to the sediment and microbial mats. Green nodes represent the sequences in the CRISPR database with the family level assignments. All the sequences here were used from the CRISPR database with known motif level assignments. **(H)** Alignments between the consensus repeat sequences generated for specific CRISPRs motif and the repeat sequences identified in microbial mat and sediment samples.

After characterizing different CRISPR types based on unique repeat sequences, we classified these unique CRISPRs based on key binding motif (present within the repeats) for Cas endoribonucleases, which play a significant role in the adaptive immunity conferred by CRISPR cassettes (Lange et al., 2013). Cas endoribonucleases bind to CRISPR repeat sequences for identification and inhibition of attacks by phages. The binding affinity of Cas proteins is dependent on a key binding motif present in the repeat sequence, and here we have characterized the reconstructed CRISPR cassettes based on these motifs 1–33 (Lange et al., 2013). Out of 87 different CRISPR cassettes identified across both microbial mat and sediment metagenomes, only 24 were assigned with these motif types—just 28% of the repeats showed matches in the existing database, with the 72% of CRISPR arrays being novel (Figures 3, 4). These results again highlight the limited database that exists for viruses overall and how much is still unknown and uncharacterized.

### Metagenomic Reconstruction of Potential Bacteriophage Hosts

**FIGURE 5:**
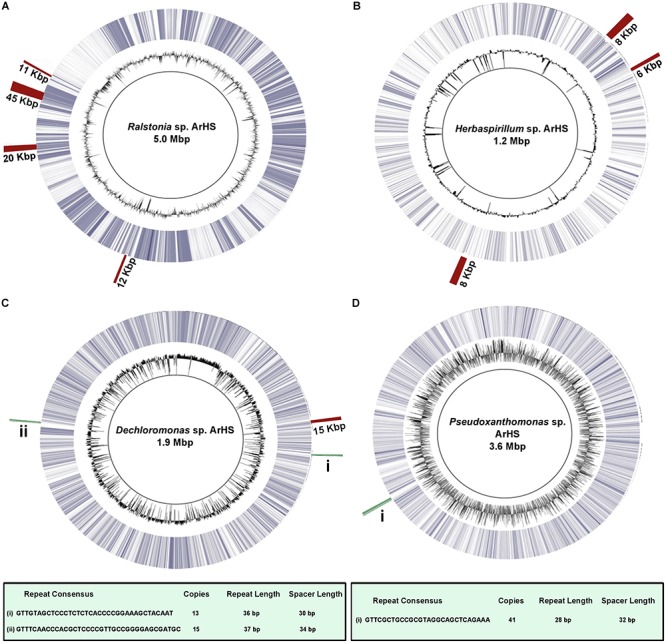
Circular alignments of reconstructed genotypes of **(A)** *Ralstonia* sp. ArHS, **(B)** *Herbaspirillum* sp. ArHS, **(C)** *Dechloromonas* sp. ArHS, **(D)** *Pseudoxanthomonas* sp. ArHS with respect to their closest reference genotypes, i.e., *Ralstonia solanacearum* GMI1000, *Herbaspirillum seropedicae* SmR1, *Dechloromonas aromatica* RCB, *Pseudoxanthomonas suwonensis* 11-1. A near complete genome (5.04 Mb, contigs = 255, reads = 2,81,07,606) of *Ralstonia* sp. ArHS was recovered based on the conserved bacterial genes [31/31 single copy genes (Wu and Scott, 2012) and 106/107 essential genes (Dupont et al., 2012)]. However, only partial genomes were recovered for *Herbaspirillum* sp. ArHS (1.2 Mb), *Dechloromonas* sp. ArHS (1.9 Mb), and *Pseudoxanthomonas* sp. ArHS (3.5 Mb) with less than 95% of conserved genes. From center toward outside; Ring 1 (solid black line): reconstructed bacterial genome, Ring 2 (black): G+C content, Ring 3 (blue): bacterial reference genome, and Ring 4: homologous region between prophages (red) or CRISPR arrays (green) and the reconstructed bacterial genome.

Bacterial diversity analyses (based on essential genes identified using MetaPhlAn) of sediment and microbial mat samples revealed an abundance of genera like *Ralstonia, Burkholderia, Enterobacteria, Herbaspirillum*, etc. Draft genomes of four potential phage hosts, i.e., *Ralstonia* (5 Mb), *Pseudoxanthomonas* (3.5 Mb), *Dechloromonas* (1.9 Mb), and *Herbaspirillum* (1.2 Mb) were reconstructed from microbial mats (MM2) (Figure 5). Metagenomic fragment recruitment of MM2 samples also revealed significant coverage for the reference genomes *Dechloromonas aromatica* RCB (*n* = 32,64,075), *Herbaspirillum seropedicae* SmR1 (*n* = 71,57,193), *Pseudoxanthomonas suwonensis* 11-1 (*n* = 35,05,890), and *Ralstonia mannitolilytica* (*n* = 70,30,087) (Supplementary Figure S4). A near complete genome (5.04 Mb, contigs = 255, reads = 2,81,07,606) of *Ralstonia* sp. ArHS was recovered and further characterized (*P*-value < 0.001) at the genus level by MetaBin (relative abundance, 8.5%) (Sharma et al., 2012). *Ralstonia* sp. ArHS was annotated with 4,819 coding sequences. Genome-wide analysis revealed the presence of four prophages with sizes 12.2 kb, 20.7 kb, 44.8 kb, and 11.2 kb (Figure 5 and Supplementary Figure S5). Interestingly, the CRISPR-associated gene *cas1* (which is generally present upstream or downstream of CRISPR array) was found in the genome of strain ArHS, however, we were not able to identify intact CRISPR array in the genome. The identification of prophages in the genome suggests that *Ralstonia* sp. ArHS is a phage host in the microbial mats of the Manikaran hot springs (Rath et al., 2015).

Partial genomes of *Dechloromonas* sp. ArHS (1.9 Mb) and *Herbaspirillum* sp. ArHS (1.2 Mb) were screened for prophages and CRISPR arrays. One prophage (13.5 kb) was identified in *Dechloromonas* sp. *ArHS* and three prophages (8 kb, 6 kb, and 8 kb) were identified in *Herbaspirillum* sp. ArHS. Further, composition-based analysis of these arrays revealed two distinct types (based on repeat sequences) in *Dechloromonas* sp. ArHS, with 13 and 15 copies across the genome (Supplementary Table S3 and Supplementary Figures S6, S7). Similarly, *Pseudoxanthomonas* sp. ArHS showed 41 copies of an array with unique repeat type (Supplementary Figure S7). The presence of intact CRISPR arrays in the reconstructed genotypes of *Dechloromonas* and *Pseudoxanthomonas*, specifically short fragments-spacers, suggests prior frequent phage infection and acquired immunity against the same phage over time.

## Conclusion

We employed metagenomics and electron-ion microscopy to study viral diversity at the Manikaran hot springs in India. We investigated viral enrichment cultures produced from sediment and microbial mat samples, which revealed the presence of archaeal viruses belonging to family *Fuselloviridae* with varying sizes within sediments, and tailed or tailless phages within microbial mats. Further, we used metagenomic data from microbial mat (*n* = 2) and sediment (*n* = 4) samples to reconstruct 65 bacteriophage genomes and 4 potential phage hosts. Combined, these genomes reveal potential predator–host interactions at the Manikaran hot springs using functional annotations of both viral and bacterial genomes. Both sediment and microbial mat phage genomes showed presence of both phage and host-associated genes suggesting potential lateral gene transfer activity between these phage genomes and their hosts in this extreme environment. This study has elucidated the prokaryotic virosphere at the Himalayan hot springs of Manikaran, providing a glimpse of the ecological roles and community dynamics of viruses, bacteria and archaea in geothermal environments. The genetic information gained in this study can be used to guide microscopy investigations (for example, with FISH probes), as well as direct isolation and cultivation efforts of Manikaran ecotypes. We provide all the data presented here as a resource for future virome studies of extreme thermophilic environments.

## Materials and Methods

### Sample Collection and Metagenome Assembly

Microbial mat and sediment samples were collected from Manikaran hot springs on 27th October, 2014 (see Supplementary Figure S1). Metagenomic DNA was isolated according to Sangwan et al., 2015). Paired-end reads were generated using an Illumina HiSeq 2000 (*n* = 42,418,084; 2 kb paired-end library) at the Beijing Genome Institute, BGI, Shenzhen, Guangdong, China. For comparative metagenomic analysis, we used data from a previously sequenced microbial mat (named MM1) (Sangwan et al., 2015) as a baseline of comparison for the four sediment (MnS1, MnS2, MnS3, and MnS4) and one microbial mat sample (MM2) sequenced in this study.

### Reconstruction of Phage Genomes and Phage Comparison

Phages were recovered from the metagenome data using the approach described in Smits et al. (2015), beginning with identification of the viral load by BLASTX against phage databases^1^ (Smits et al., 2015). The contigs were initially binned on the basis of oligonucleotide composition, contig length, and %G+C content (Sangwan et al., 2015). Phage assembly is challenging due to a greater diversity than that of bacterial genomes, thus we applied an iterative assembly strategy using PRICE assembler to overcome the coverage bias (Ruby et al., 2013). After achieving nearly complete viral genomes, *k-*mer profiling (tetranucleotide frequencies) was used to link the fragments together and allow the draft optimal genome recovery (Smits et al., 2015). In order to annotate the phages, ORFs were predicted using Prodigal (Hyatt et al., 2010) followed by BLASTP against NCBI non-redundant protein collection (NR) (at E-value 1e-5). Additionally, phage genomes were checked for functional completeness by calculating the functional completeness score employed in PHAST-Enhanced Release which uses over 200,000 annotated phage genomes as reference (Zhou et al., 2011; Arndt et al., 2016). The completeness score is calculated based on (i) identification of cornerstone genes, which include key structural genes (i.e., capsid, head, tail, coat, portal, and holin), phage DNA regulation genes (such as integrase, transposase, terminase), and phage function genes (such as lysin, bacteriocin) (Casjens, 2003), and (ii) identification of genes present in already known phage in reference database (refer to Zhou et al., 2011 and Arndt et al., 2016 for details on completeness score calculation criteria). Phage genomes were compared using a dot-plot based on pairwise whole genome sequences in Gepard_1.30 program (Krumsiek et al., 2007). Core genes were calculated between sediment and microbial mat metagenomes by using the pairwise reciprocal smallest distance (RSD) algorithm at an *E*-value and divergence cut-off of 1e-15 and 0.5, respectively (Wall and Deluca, 2007). Phage genomes were analyzed for the lifestyle [i.e., virulent (lytic) or temperate] using Phage Classification Tool Set (PHACTS) version 0.3 program employing a supervised Random Forest algorithm for classification of phage ORFs with a probability cut off value of 0.05 (McNair et al., 2012). In order to assign functionality to the phage metagenomes, we performed a HMMER 3.1b (Finn et al., 2011) search against the Caudovirales orthologous gene HMM profile^2^ downloaded from the EggNOG 4.5 database^3^.

In order to screen mat and sediment samples for archaeal viruses, we downloaded complete genomes of archaeal viruses (*n* = 95) from NCBI^4^. BLASTX was used to screen the metagenome contigs against the archaeal database. Using Bioperl script (NCBI Blast Parser ^5^), the BLASTX output was then parsed to retrieve the Best BLAST Hit (BBH) for each contig based on E-value, bit score and coverage cut-off of 80%.

### Helium-Ion Microscopy Analysis of Sediment and Mat Samples for Bacterial Diversity

Microbial mat and sediment samples were surveyed under high-resolution scanning HIM in order to investigate the bacterial diversity in each sample. In preparation of microscopic analysis, samples were dehydrated by increasing ethanol concentration steadily to 70% within an hour. At this point, the samples were stored in the fridge at 4°C for 24 h so as to exploit the gentle fixative effect of ethanol on cell walls. After 1 day, the ethanol concentration was increased to 100%. Next, the samples were dried in a fully automated Leica CPD300 (Austria) critical point dryer, after which the samples were mounted on standard SEM stubs using a silver-epoxy.

The HIM was carried out with a Zeiss (United States) ORION nanoFab helium-ion microscope. The ion energy and beam current amounted to 25 kV and 0.6 pA, respectively. For the imaging, we employed the Everhard-Thornley secondary-electron detector.

### Virus Enrichment Followed by Electron Microscopy

A mass of 5 g from each of the microbial mat and sediments samples was deposited into 50 mL falcons and 0.1 M ammonium acetate was added until a total volume of 45 mL was reached. The samples were then placed on a shaker for 2 h and kept at room temperature in an incubator. Following that, the tubes were centrifugalized at 10,000 × *g* for 20 min at 4°C. The supernatant was filtered to remove cells and cell debris using 0.22 μm syringe filters (Merck Millipore, Germany). The filtrate was stored at 4°C until downstream processing. Virus concentration and purification was carried out with two aliquots (5.5 mL in polyallomer tubes) of each sample in a table-top ultracentrifuge Optima MAX-XP (Beckman Coulter, United States) equipped with a swinging bucket rotor MLS-50 (Beckman Coulter, United States). The samples were spun at 80,000 × *g* at 4°C under vacuum conditions for 4 h. The supernatant was discarded and 50 μL of 0.1 M ammonium acetate (pH 7.0) was added to each centrifuge tube to re-suspend the virus pellet and left overnight.

Afterward, 10 μL aliquots of the virus preparations were placed onto parafilm, onto which a 300-mesh formvar-coated copper grid (Plano, Germany) was placed (film down) and left to sit for 1 min in order to allow the viruses to attach to the grid. The excess liquid was blotted off the surface of the grid, and the dried grid was stained twice with a 2% uranium acetate solution, for 30 s each time. The grid was left to dry in a petri-dish overnight, after which the samples were analyzed using a Zeiss (Germany) Merlin VP compact SEM with an option of transmission imaging.

### Environmental CRISPR Identification and Characterization

Clustered Regularly Interspaced Short Palindromic Repeats were identified in the metagenome samples by using PILER-CR v1.06 (Edgar, 2007) and CRISPRFinder (Grissa et al., 2007). CRISPR arrays were assigned taxonomic status by using contigs and CRISPR repeats through a BLASTx search against NCBI non-redundant protein collection (NR) with an E-value 10^-5^. For both approaches, taxonomic status was assigned if the top 10 hits belonged to one phylum; taxonomic labels at the level of class, family, and genus were assigned if the majority of the top 30 hits belonged to the same taxon of that level. If the top 10 labels for that specific contig or repeat were relatively diverse, then no status was assigned. Moreover, the CRISPR cassettes were characterized at the motif (1–33) and family (1–40) levels based on a repeat consensus using CRISPRMap (Lange et al., 2013).

### Taxonomic Binning for Potential Phage Host Identification

Microbial mat (*n* = 2) and sediment (*n* = 4) metagenomes were binned for phylogenetic composition using MetaBin (Sharma et al., 2012), MetaPhlAn (Segata et al., 2012), and BLASTx bit-score with a Lowest Common Ancestor (LCA) approach. The contigs (>10 kb) were clustered in R4.3.2 on the basis of tetranucleotide frequency, coverage, and %G+C content to generate multiple bins at each parameter^6^. Broadly, four distinct bins were generated based on tetranucleotide frequency and coverage, to which they were further binned for taxonomical assignments and revealed four independent genera—*Ralstonia* (5.1 Mb), *Pseudoxanthomonas* (3.5 Mb), *Dechloromonas* (1.9 Mb), and *Herbaspirillum* (1.2 Mb). Finally, each bin was then analyzed for completeness through validation using 107 essential (Segata et al., 2012) and 31 bacterial marker genes (Wu and Scott, 2012) leading to a result of 99.9% completeness for *Ralstonia* sp., which was then labeled *Ralstonia* sp. ArHS.

## Availability of Data and Materials

Metagenome sequence data for microbial mats was deposited under accession numbers: MM1-PRJEB4614 (http://www.ebi.ac.uk/ena/data/view/PRJEB4614) and MM2-PRJEB14856 (http://www.ebi.ac.uk/ena/data/view/PRJEB14856) in EBI. All the sediment samples (MnS1, MnS2, MnS3, and MnS4) were submitted to DDBJ/EMBL/GenBank under the accession number: PRJEB14843 (http://www.ebi.ac.uk/ena/data/view/PRJEB14843), respectively. The metagenome data for MM2 were submitted under the accession number PRJEB14856. The phage genomes recovered from microbial mats and sediments were deposited under the accession numbers ERS2768334-ERS278398 under the project PRJEB14843.

## Author Contributions

RL developed the concept for the study. RL and AS performed the metagenomic analyses. AS, MS, BK, and NM did the viral enrichments and analyzed the samples under ion/electron microscopes. RL, AS, NM, LC, YS, HR, WA, and JG wrote the manuscript.

## Conflict of Interest Statement

The authors declare that the research was conducted in the absence of any commercial or financial relationships that could be construed as a potential conflict of interest.

## Footnotes

1 http://www.phantome.org/Downloads/

2 http://eggnogdb.embl.de/download/eggnog_4.5/data/viruses/Caudovirales/

3 http://eggnogdb.embl.de/#/app/viruses

4 https://www.ncbi.nlm.nih.gov/genomes/GenomesGroup.cgi?taxid=10239

5 http://www.bioinformatics-made-simple.com/2012/07/ncbi-blast-parser-extract-query-and.html

6 http://www.R-project.org/
