# Supplementary material for: Bacterial and Archaeal Viruses of Himalayan Hot Springs at Manikaran Modulate Host Genomes
Source: Front Microbiol. 2018 Dec 14;9:3095. doi: 10.3389/fmicb.2018.03095 (PMC6302217; doi:10.3389/fmicb.2018.03095)
Supplement: Supplementary file 1 [file Data_Sheet_1.PDF]

Bacterial and Archaeal Viruses of Himalayan Hot Springs at Manikaran modulate Host Genomes

Anukriti Sharma[1,2,3], Matthias Schmidt[4], Bärbel Kiesel[5], Nitish K. Mahato[1], Lauren Cralle[2,3], Yogendra Singh[1], Hans H. Richnow[4], Jack A. Gilbert[2,3], Wyatt Arnold[3], and Rup Lal[1*]

Department of Zoology, University of Delhi, Delhi, India[1]

Biosciences Division (BIO), Argonne National Laboratory, 9700 South Cass Avenue, Argonne, IL, U.S.A.[2]

Department of Surgery, University of Chicago, 5841 S Maryland Ave, Chicago, IL, U.S.A.[3]

Department of Isotope Biogeochemistry, Helmholtz Centre for Environmental Research, Leipzig, Germany[4]

Department of Environmental Microbiology, Helmholtz Centre for Environmental Research, Leipzig, Germany[5]

Corresponding author[#]

Correspondence and requests for materials should be addressed to R.L (email: ruplal@gmail.com).

Running Title: Prokaryotic virosphere at Manikaran hot springs

Supplementary Figures

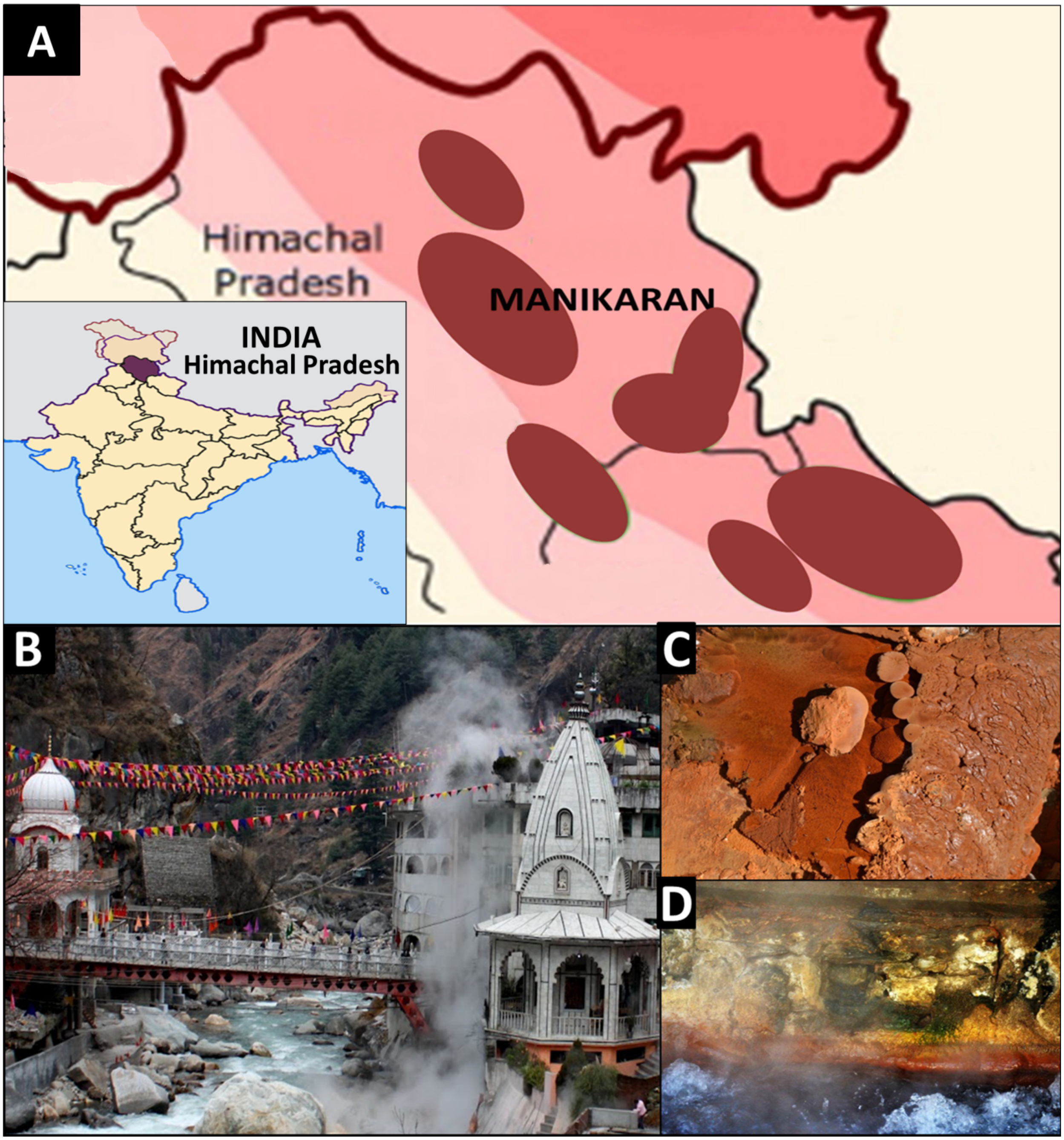

Figure S1: (A) Geothermal fields (shown in red ovals) of Himachal Pradesh, India with the highest temperature source at Manikaran (>95°C)**. (B)** The Manikaran temple which encloses the sampling site for **(C)** sediment and **(D)** microbial mats used for analyses in this study.

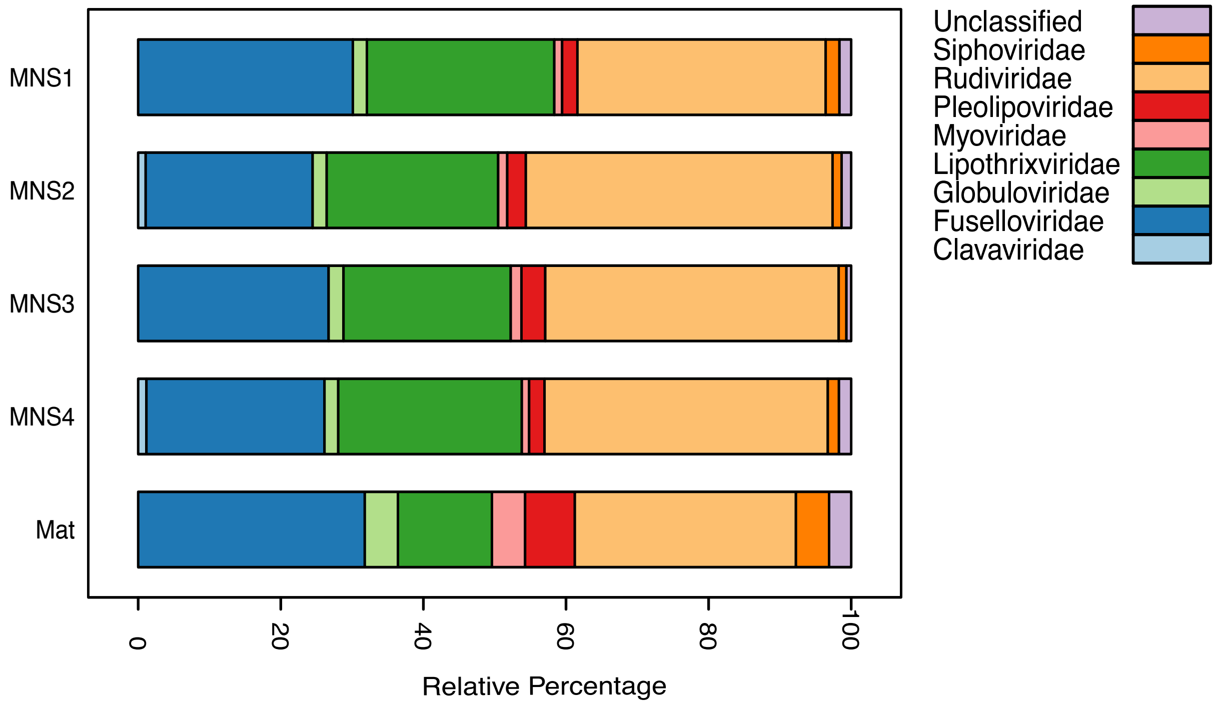

Figure S2: Distribution of eight different archaeal virus families across different sample types i.e. microbial mats and sediments.

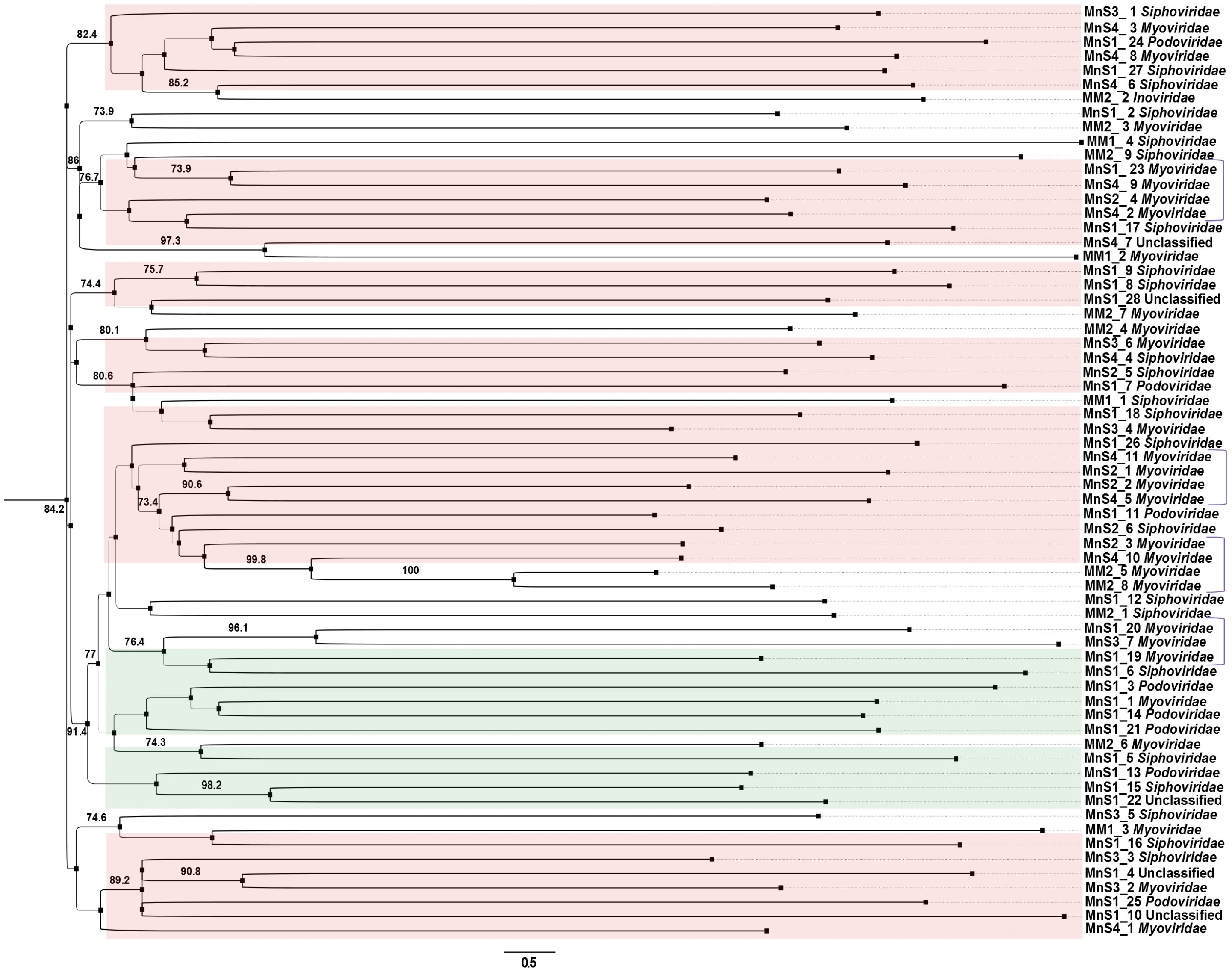

Figure S3: Phylogenomic reconstruction of phage genomes based on complete predicted proteome. Bootstrap values above 70% are labelled. Clades highlighted in light green color demonstrate site-specific clustering for MnS1 samples. Clades highlighted in pink color show clustering pattern between rest of the sediment samples i.e. (MnS2, MnS3, and MnS4).

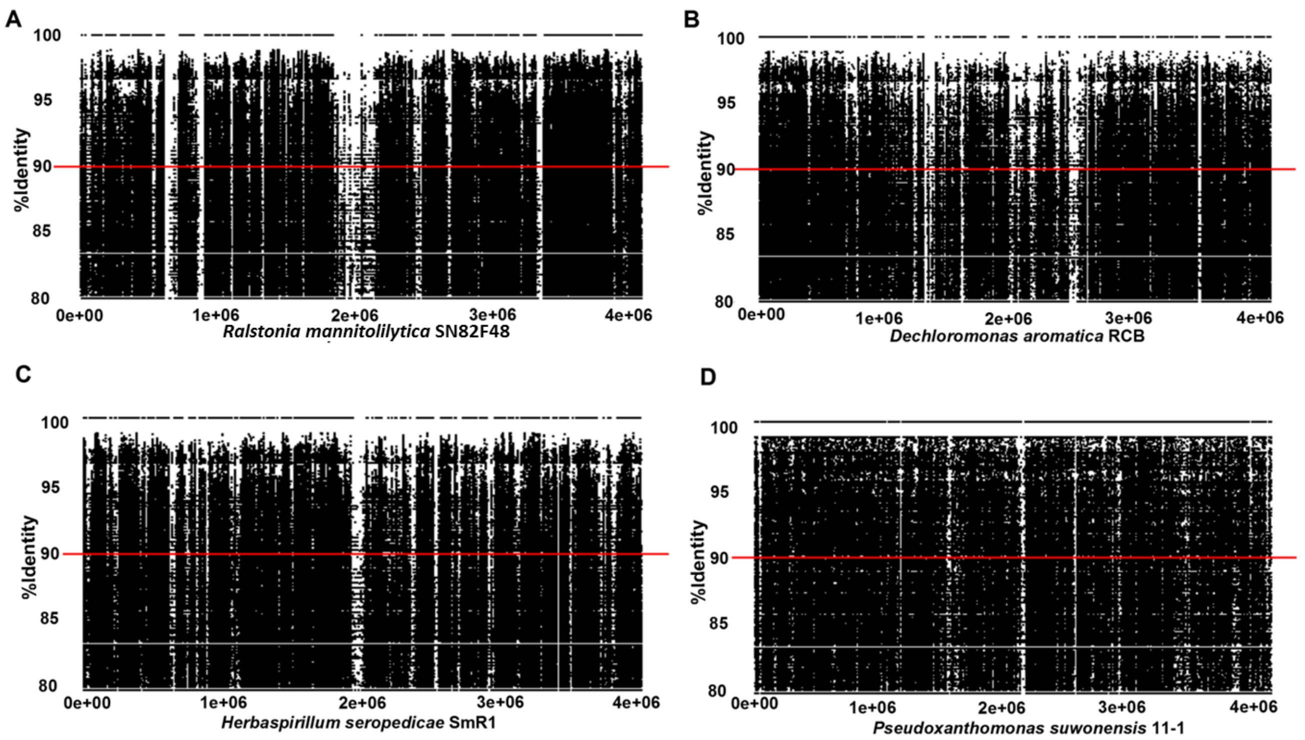

Figure S4: Metagenomic fragment recruitment of enriched microbial mat on the reference genomes. (A) *Ralstonia mannitolilytica* SN82F48, (B) *Dechloromonas aromatic* RCB, (C) *Herbaspirillum seropedicae* SmR1, and (D) *Pseudoxanthomonas suwonensis* 11-1.

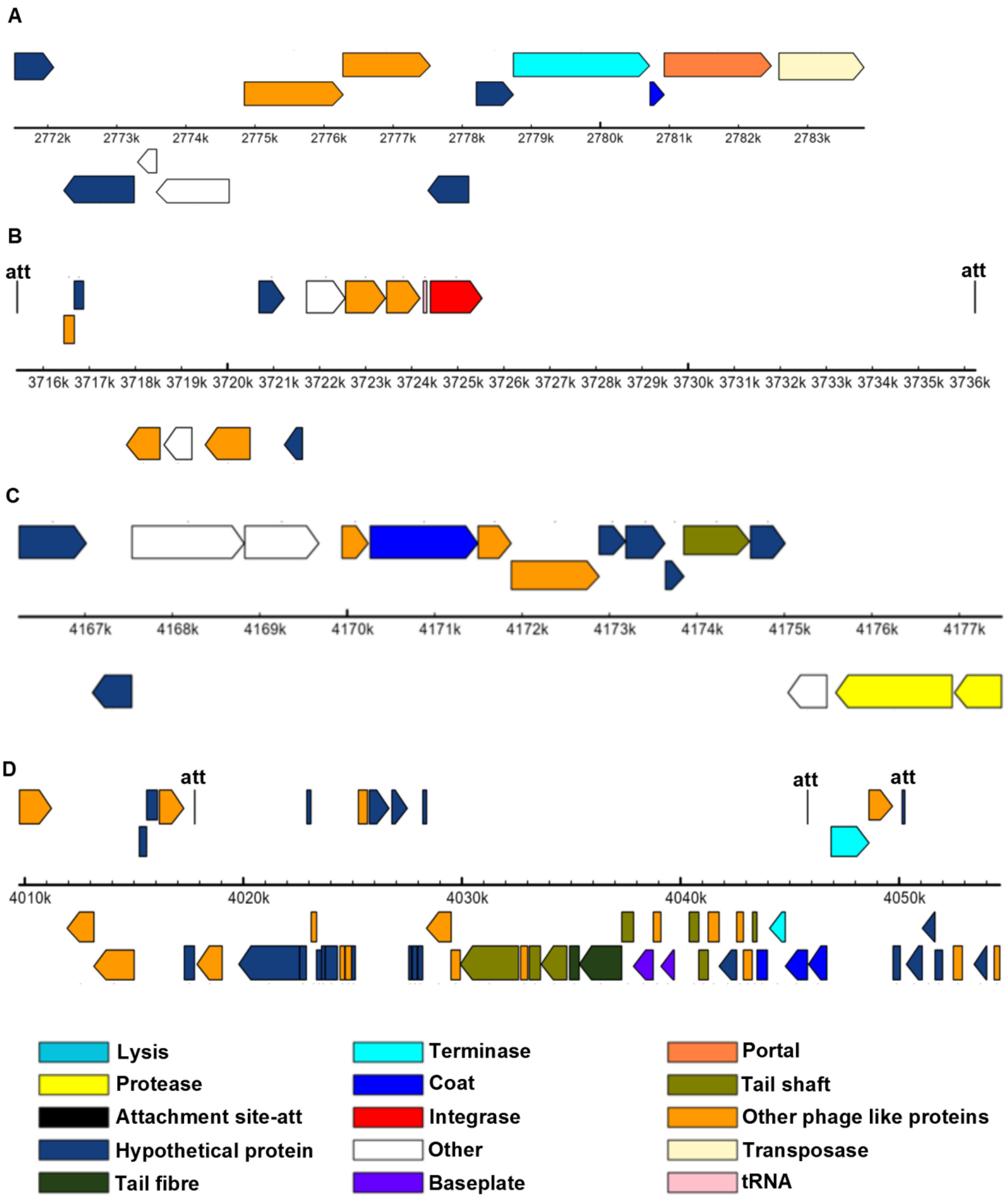

Figure S5: (A-D) Schematic representation of four phages reconstructed from the genome of *Ralstonia* sp. ArHS with index for the color coding of ORFs in the last panel.

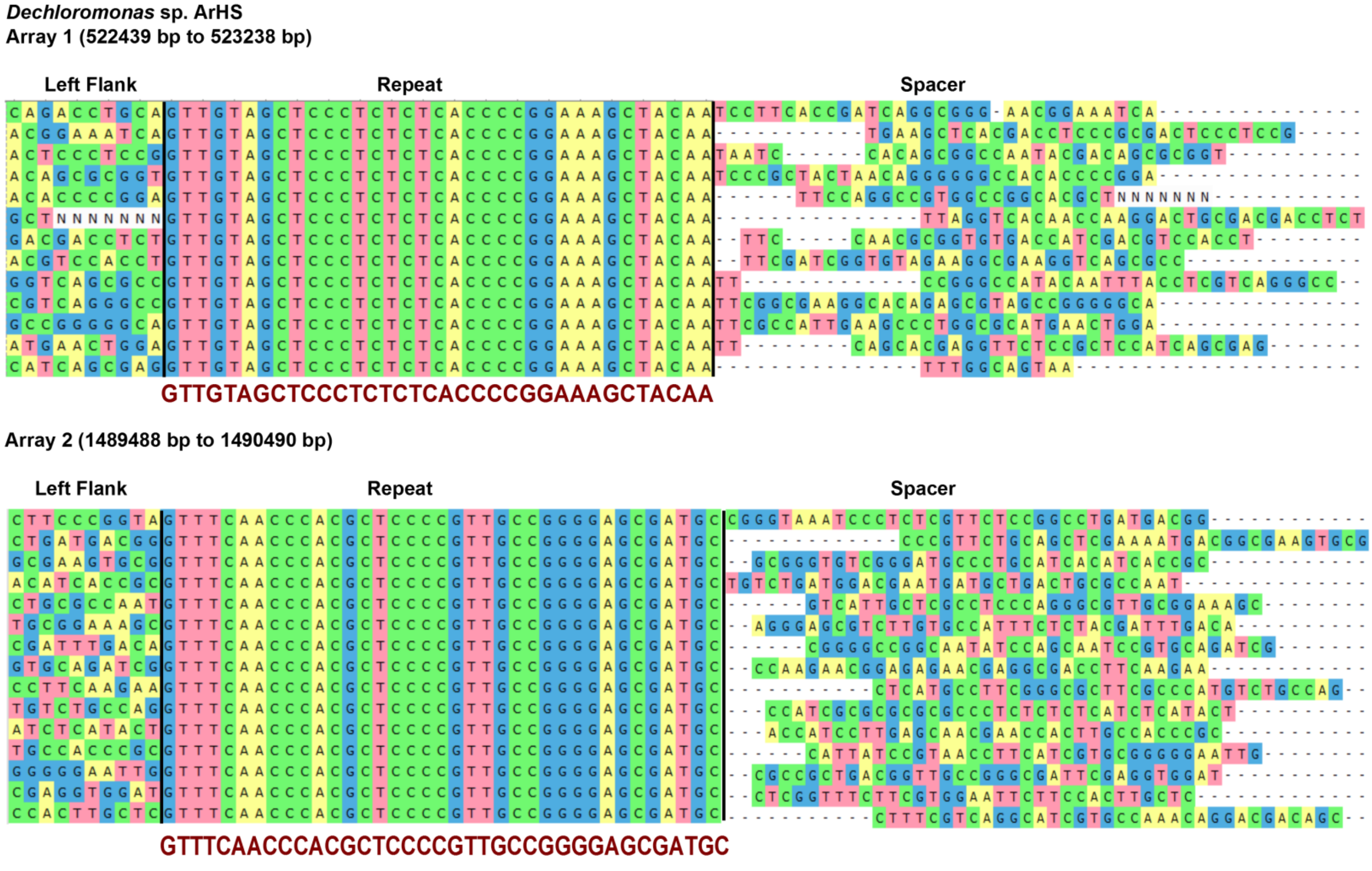

Figure S6: Schematic representation of two types of CRISPR arrays determined in *Dechloromonas* sp. strain ArHS.

***Pseudoxanthomonas* sp. ArHS**
Array 1 (2386538 bp to 2398696 bp)

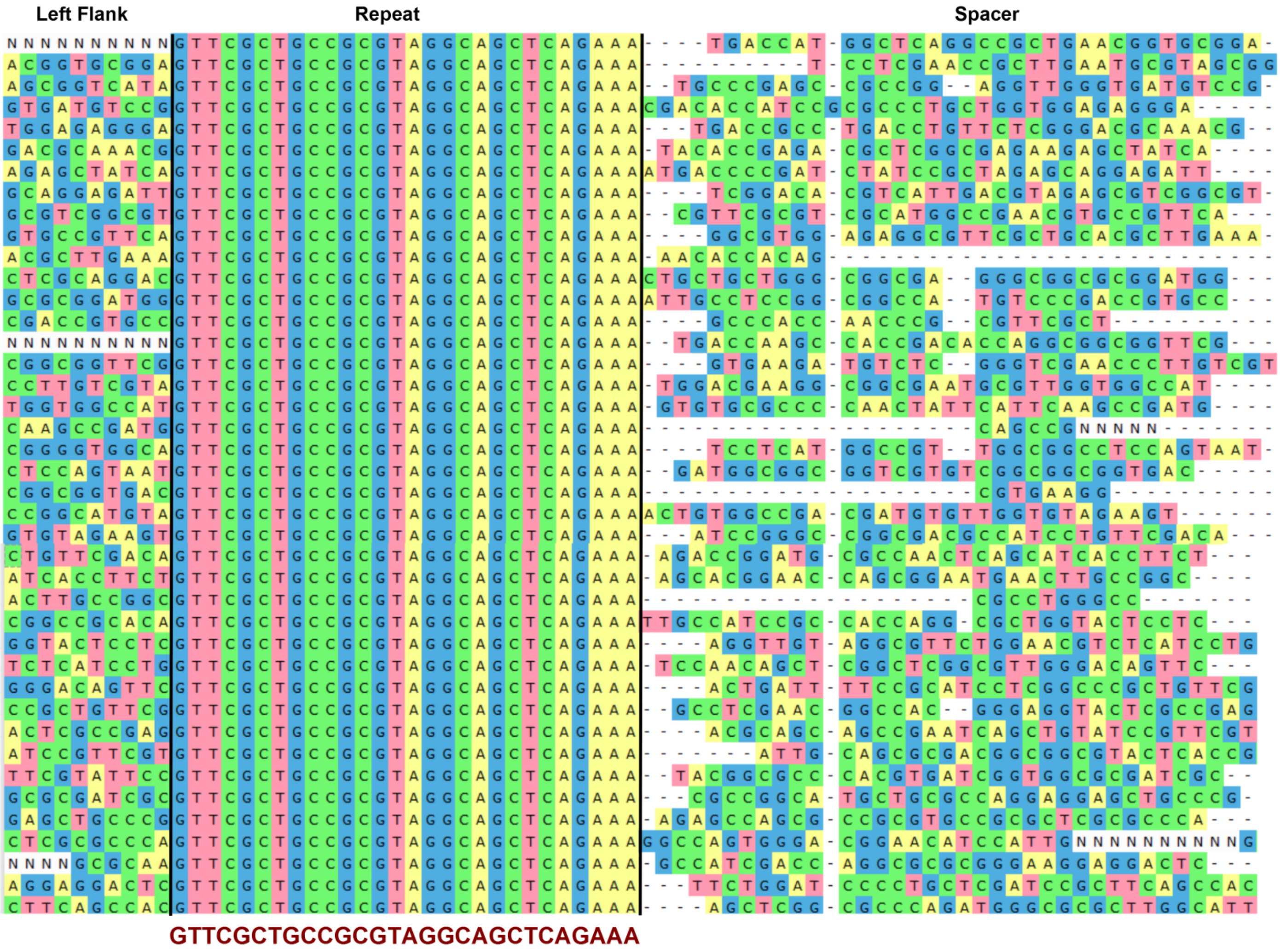

Figure S7: Schematic representation of one type of CRISPR array determined in *Pseudoxanthomonas* sp. ArHS.

Supplementary Tables

Table S1: List of eight functional categories created from 99 orthologous genes (OGs) of *Caudovirales*.

S. No.	Functional Category	OGs
1.	Host lysis	Bacteriophage Rz lysis protein<br>Cell wall macromolecule catabolic process<br>Gene 25-like lysozyme<br>Peptidase S24-like<br>Peptidoglycan metabolic process<br>Exonuclease activity<br>Cobalamin binding<br>Peptidase family S49<br>Nuclease activity<br>Peptidase activity<br>Glycosyl hydrolase 108<br>[formate-C-acetyltransferase]-activating enzyme activity
2.	Replication	DNA-directed RNA polymerase N-terminal<br>Macro domain<br>Bacteriophage replication gene A protein (GPA)<br>DNA polymerase family A<br>ATP binding<br>DNA ligase (ATP) activity<br>DNA binding<br>Ribonucleotide reductase N-terminal<br>DNA-binding protein<br>DNA polymerase III alpha subunit<br>Ribonucleotide reductase, all-alpha domain<br>deoxyribonucleoside diphosphate metabolic process<br>Helicase conserved C-terminal domain<br>RNA ligase<br>Thymidylate synthase (FAD) activity<br>DNA gyrase/topoisomerase IV, subunit A<br>5'-3' exonuclease, C-terminal SAM fold<br>NAD+ binding
3.	Recombination	NinF protein<br>T4 recombination endonuclease VII, dimerization<br>Sequence-specific DNA binding<br>NinB protein<br>Bacteriophage Lambda NinG protein<br>Recombination endonuclease VII<br>Recombinase activity<br>Phage Mu protein F like protein<br>Phage NinH protein<br>Histidine kinase-, HSP90-like ATPase
4.	Structural	Caudovirales tail fibre assembly protein<br>Bacteriophage head to tail connecting protein<br>Phage late control gene D protein (GPD)<br>Phage tail fibre repeat

		Baseplate J-like protein<br>T7 icosahedral viral capsid
5.	Morphogenesis	Virion<br>HNH endonuclease<br>Establishment of integrated proviral latency<br>Metal ion binding<br>Phage small terminase subunit<br>Phage portal protein<br>Phage capsid scaffolding protein (GPO) serine peptidase<br>Phage portal protein, lambda family<br>Phage major capsid protein, P2 family<br>Putative ATPase subunit of terminase (gpP-like)<br>ParB-like nuclease domain<br>dUTPase<br>Phage DNA packaging protein Nu1
6.	Lysis-Lysogeny switching	Phosphoprotein phosphatase activity<br>Kinase activity<br>Clp protease<br>Bacteriophage CII protein
7.	Restriction of host peptides	Quinone binding<br>D12 class N6 adenine-specific DNA methyltransferase<br>C-5 cytosine-specific DNA methylase<br>Methyltransferase activity<br>DNA N-6-adenine-methyltransferase (Dam)<br>Flavin adenine dinucleotide binding
8.	Hypothetical proteins*	27

*In case of hypothetical proteins, only the total number is mentioned

Table S2: List of CRISPR cassettes identified across microbial mat and sediment samples of Manikaran hot springs.

Metagenome specific repeat type	No. of CRISPR Arrays	Average Repeat Length	Average Spacer Length	Repeat Consensus	CrisprMap Structure	Crispr Map Family	Crispr Map Superclass	Phylum Assigned
MM2_1	3	37	35	GTTTCAATCCACGTCCGGCATCACAGCCGGACGACAC	0	family 3	D	Proteobacteria
MM2_2	2	37	30	**O**ATTGTAGCTTTCCGGGGTGAGAGAGGGAGCTACAAC	0	0	E	Proteobacteria
MM2_3	1	16	33	CCACGCTCGAAAAACG	0	0	F	Proteobacteria
MM2_4	1	37	34	GCATCGCTCCCCGGCAACGGGGAGCGTGGGTTGAAAC	0	family 3	D	Proteobacteria
MM1_1	6	37	34	**WY**T**V**AAA**MM**TTGCCCTGATGAAGAAGGGATTAAGAC	0	0	E	Proteobacteria
MM1_2	5	38	35	GTTTCTATTCGCTGTGACGCGAAGGGTG**Y**TCCGGAAC	0	0	E	Proteobacteria
MM1_3	3	28	32	GTTCRCTGCCGTAYAGGCAGCTYAGAAA	motif 4	0	C	Proteobacteria
MM1_4	3	30	36	CTTTTAATCGAACTAATGTAGAATTGAAAC	0	family 25	A	Bacteriodetes
MM1_5	3	35	36	TCAAAACTTCTTCGGAAGTTGAATTAATGGAAAC	0	0	D	Cyanobacteria
MM1_6	3	37	37	GTTTCCAGCCCCTTCAAAGCGGGGCGCCGATCCTCAC	0	0	E	Bacteriodetes
MM1_7	3	35	36	GTGCTCAACGCCTAACGGCATCAAAGGTAATTCAC	motif 5	family 39	E	Spirochetes
MM1_8	2	29	32	CTTCTAAACCGCCTACACGGCGGGAAAC	motif 4	family 5	C	Chloroflexi
MM1_9	2	37	36	GTTTCAGAGCTCAATCGTGAGCCGATGATCGTGCAAT	0	0	E	Actinobacteria
MM1_10	2	30	36	ATATGAATCGAACCTCTAAGGAATTGAGAC	0	0	A	Not assigned
MM1_11	1	37	35	AAGGCTTTACCATC	0	0	E	Firmicutes

				GGCTCGGGGTTGAG CACTGAAAC				
MM1_12	1	37	36	GTTTCAGTCCCGTT GCCGGGATTCAAGT ATTGGCGAC	motif 30	0	D	Cyanobacteria
MM1_13	1	36	36	GTCCCCACTCGCTG GGGAAAACAATTG AATGGAAAC	0	0	E	Cyanobacteria
MM1_14	1	36	32	GTTTCAATCGCCTG ATTTGGCGCAATGC CCTATAGG	0	0	E	Proteobacteria
MM1_15	1	36	37	GCTTCAATTGAGCC ACGCTTATTCAGCG TGGAGAAC	0	0	D	Eukaryoarcha eta
MM1_16	1	37	36	GTGACCAACCCCTT CAGAGCGGGGCTG TGATCGGCTG	motif 24	0	E	Actinobacteria
MM1_17	1	28	36	CTTTCTACTCCCTA TGGGAAACATAAA C	0	0	A	Bacteriodetes
MM1_18	1	37	37	CTTGGGAGGCAGG GGAATCCTCGGAG AGGACTGAAAC	0	family 11	E	Proteobacteria
MM1_19	1	37	36	GTTGCAGCGGCCTC TGCATGGCCGATGG CAATGGAAG	motif 24	0	D	Proteobacteria
MM1_20	1	36	29	GTTGTGGTTTGATT AAAGATTAGAAAA CACGATATT	0	0	E	Not assigned
MM1_21	1	36	29	GTTGGGGCGTCCGG TCAGACCCCTTTCT GATACACT	0	0	D	Euryarchaeta
MM1_22	1	37	36	CTTCCACTGGTCGT TACACGAAATTGGT CACTGCAAC	0	0	D	Not assigned
MM1_23	1	36	35	GCTTCAATGGGGCC GTGCCTTCTCAGGC ACGGAAAG	motif 23	0	E	Euryarchaeota
Mns2_1	225	24	41	OGWMGATCTDCTA TAGAATTGAAAG	0	family 11	F	Crenarchaeota
Mns2_2	12	24	40	KRATCYCGMGAOA GRGAGTTGAAAG	0	0	F	Actinobacteria
Mns2_3	12	25	43	CYAGAAATCAAAA GATAGTTGAAAC	0	0	F	Crenarchaeota
Mns2_4	12	28	32	CTTCACTGCCGAAC AGGCAGCTTAGAA A	motif 4	family 5	C	Proteobacteria
Mns2_5	11	24	44	GAATCTCAAAAAG AGGWTTGAAAG	0	family 11	F	Crenarchaeota
Mns2_6	8	29	32	GTTCTCCCCGCGCA	0	family	C	Actinobacteria

				GGCGGGGGTGGTC C		33		
Mns2_7	7	29	37	GTTTTATCTGAACT ATGTGGGATGTAAA G	0	family 6	A	Acquificae
Mns2_8	7	24	35	CTTTTAGTTCTTTGT TATTTWRWG	0	0	F	Not assigned
Mns2_9	5	32	34	GTTTCAATCCACGC GCCTGCGTGAGGCG CGAC	motif 3	family 3	D	Proteobacteria
Mns2_10	3	36	39	GTTGCGGTTGGCCT GCGATTTCTGAACT GCTAGGCC	0	0	E	Proteobacteria
Mns2_11	2	46	30	GCTGTGATTCCCTT GGGAAGATATCAA TTTGAAAGCAATTC ACAAC	0	0	0	Bacteriodetes
Mns2_12	1	23	50	GAGTAGCACCGAC CGGGCGAAAG	0	0	F	Not assigned
Mns2_13	1	21	54	AGTTACGTTCGATG TATTCCA	0	0	F	Proteobacteria
Mns2_14	1	19	36	TCGAGAGCGTGAG CGGCGA	0	0	F	Proteobacteria
Mns3_1	10	36	36	ATTTCCGCGRYYRA WWKWWCGCGGCC CCATTGAAGC	motif 33	0	F	Proteobacteria
Mns3_2	7	30	36	GTTTKTAGCCTRCC TRTGAGGGATTGAA AC	0	0	A	Firmicutes
Mns3_3	5	29	32	CGGTTCATCCCCGC GTGTGCGGGGAAY AG	0	family 2	B	Proteobacteria
Mns3_4	4	37	33	GTTTTGAGTGAGAC CTTATGAAGAAGG GATTGAGAC	0	family 11	E	Proteobacteria
Mns3_5	4	37	35	GTTTCAATCCTCAC CCGACCCGAAGGC CGGGTGCAAG	0	family 32	D	Proteobacteria
Mns3_6	3	37	37	CAGCAGAGCATTGC CCCGCAATGAAGG GGTTTGAAAC	0	0	E	Not assigned
Mns3_7	3	36	35	GCTTCAATTGCGCC ACGCCTAATAAGGC ATGGAAAC	0	0	D	Euryarchaeota
Mns3_8	3	38	37	GTTTCAATCCTCTC TTCGTCGAGGCAAC TSTTCTAAT	0	family 11	E	Not assigned
Mns3_9	2	28	33	GGTCCACCCCCACG TGCGTGGGGACGTC	0	family 21	C	Actinobacteria
Mns3_10	2	33	34	GTGCTAATCGAACC	0	family	A	Euryarchaeota

				AAKGTGGGATTGA AAC		6		
Mns3_11	2	37	37	GWKWCAACCKWA CGACCCGYGWTTA GGGTACTGAAAG	motif 21	0	B	Proteobacteria
Mns3_12	2	38	39	GTCGGAAGACAGT GAATCCTCGTCAGG GGATTGAGAG	motif 5	family 11	E	Actinobacteria
Mns3_13	1	36	29	GGTGGAAAGGGCC GGAGATTTACCGGC TCTGACACC	motif 29	0	E	Acidobacter
Mns3_14	1	38	38	GGTCGCAATCCTCT TTAAAGCGAGTCCT GGTTTCCGAC	0	0	E	Firmicutes
Mns3_15	1	37	37	GTTTCAGTCCCGTT GCCGGGATTTAGAG AGGTGAAAC	motif 30	0	D	Firmicutes
Mns3_16	1	42	30	GTCAGTCTCAATCC CTTGCGCAAGGGCG GTCCTTCGGGACTG	motif 32	0	E	Firmicutes
Mns3_17	1	30	35	CTTTTAATCGCACC TATTTGGAATTGAA AT	0	0	A	Bacteriodetes
Mns3_18	1	37	37	GTCGCAATCCTTGT TGTGGTGGAAGATG GGTTCTGAG	0	0	E	Bacteriodetes
Mns3_19	1	58	19	TATCACTGTTGATA AATATCCAAAACCT ATAAGTGTTGATAT ATCCAAGACATATC AA	motif 32	0	0	Not assigned
Mns3a_1	6	36	36	GTTTTGGCTCTTCG GCTCGTGGTTGAGC ACTGAAAC	0	0	E	Proteobacteria
Mns3a_2	5	37	35	GTTTCAATATCCTC TACGGATCGAAGTG AACTGAAG	0	0	E	Proteobacteria
Mns3a_3	2	37	36	GTTTCAATCCATTC CCTCTTTTCAGAGG AATTCGAC	0	0	D	Proteobacteria
Mns3a_4	2	36	37	GTTTCCACGTTCGG CTGAGCGTGGCTCC ATTGAAGC	0	0	0	Euryarchaeota
Mns3a_5	1	36	34	GCGATTTTTCCGGT GATGCCGCGAGGC GTTGAGCAC	motif 5	0	E	Proteobacteria
Mns3a_6	1	37	36	GTTCGAAAAGCAA AAAATCCGCACGG CGGATTGAAAC	0	0	E	Oscillochloris trichoides/Chl oroflexi
Mns4_1	11	37	36	CTTCAATGGGGCCG	0	0	F	Proteobacteria

				CRVYHDVRWKRBY GCGO				
Mns4_2	9	37	37	TYGCACCSGGYYW YBGGYYWYCGRGC CSGGTGAGGRTTGA AAC	motif 3	0	F	Oscillochloris trichoides/Chl oroflexi
Mns4_3	9	37	36	GTTCGAAAAGCAA AAAATCCGCACGG CGGATTGAAAC	0	0	E	Firmicutes
Mns4_4	7	37	35	CTTCAGTTCACTTC GATCCGTAGAGGAT ATTGAAAC	0	0	E	Not assigned
Mns4_5	7	37	36	RHHKCAVCBCDWC GATCCGMBWARGG GATACTGAAAG	0	0	F	Crenarchaeota
Mns4_6	6	36	36	GTTTTGGCTCTTCG GCTCGTGGTTGAGC ACTGAAAC	0	0	E	Proteobacteria
Mns4_7	5	37	36	GTTTCAATCCCCAG CCGTCCCGAAGGAC GGCTGCTAC	0	family 32	D	Not assigned
Mns4_8	3	36	42	GTTTCAGTCCCCCT CGGGGGATTTTTC GCGTCTAG	motif 5	family 11	E	Not assigned
Mns4_9	3	36	37	GTCTCCACGSYYKM ARKRSCGTGGCCCC ATTGMRO	0	0	F	Euryarchaeota
Mns4_10	2	37	36	GTTTCAATCCATTC CCTCTTTTCAGAGG AATTCGAC	0	0	D	Planctomycete s
Mns4_11	2	38	38	GATTCAGCTTGATG CCCCGCAGATAGG GGACTGAAAC	0	0	E	Proteobacteria
Mns4_12	2	36	36	GCTTCAATGGAGCC ACGTCCTAGAGGAC GTGGAAAT	motif 23	0	E	Euryarchaeota
Mns4_13	2	37	37	GTCGCARYRGAAC GCCCGMSKMYCAG GGCATGGAAAC	motif 21	0	A	Proteobacteria
Mns4_14	2	37	38	OTRGMRMGMGCO MYGCCCCGATOGG GGATTGAAAC	0	0	F	Crenarchaeota
Mns4_15	1	37	37	GTTGAAGGGGATTT ACGGGCGAAGCGC CCATTGAAAC	motif 5	0	E	Firmicutes
Mns4_16	1	36	37	ATTTCCGCGACTAC TCAGTCGCGGCCCC ATTGAAGC	motif 23	0	E	Not assigned
Mns4_17	1	37	35	GCGAGAAGGCCTG CGGAGGGGCCTTGT	0	0	E	Not assigned

				TCTTCCGACC				
Mns4_18	1	28	33	GTCCTCCCCACGCACGTGGGGGTGAGCC	0	family 18	B	Proteobacteria
Mns4_19	1	36	35	GCGGTCAGCGGCGGCGCAGACCTGATCGACGGACAC	0	0	D	Actinobacteria
Mns4_20	1	28	32	TTTCTGAGCTGCCTACGCGGCAGCGAAC	motif 6	family 8	B	Proteobacteria
Mns4_21	1	36	37	GTATCCACGGTTATGTTTCCGTGGCCACATTGAAGC	0	0	0	Proteobacteria

Table S3: Characteristics of CRISPR arrays determined across the reconstructed genomes of *Pseudoxanthomonas* sp. ArHS and *Dechloromonas* sp. ArHS.

Array	Genome	Position	Length	Copies	Repeat length	Spacer length	Consensus
1	*Pseudoxanthomonas* sp. ArHS	2386538	630	11	28	32	GTTCGCTGCCGCGTAGGCAGCTCAGAAA
2	*Pseudoxanthomonas* sp. ArHS	2389282	148	3	28	32	GTTCGCTGCCGCGTAGGCAGCTCAGAAA
3	*Pseudoxanthomonas* sp. ArHS	2389529	268	5	28	32	GTTCGCTGCCGCGTAGGCAGCTCAGAAA
4	*Pseudoxanthomonas* sp. ArHS	2391831	150	3	30	30	GTTCGCTGCCGCGTAGGCAGCTCAGAAATG
5	*Pseudoxanthomonas* sp. ArHS	2392131	269	5	29	31	CGTTCGCTGCCGCGTAGGCAGCTCAGAAA
6	*Pseudoxanthomonas* sp. ArHS	2394135	388	7	28	32	GTTCGCTGCCGCGTAGGCAGCTCAGAAA
7	*Pseudoxanthomonas* sp. ArHS	2398326	398	7	28	33	GTTCGCTGCCGCGTAGGCAGCTCAGAAA
8	*Dechloromonas* sp. ArHS	522439	835	13	36	30	GTTGTAGCTCCCTCTCTCACCCCGGAAAGCTACAAT
9	*Dechloromonas* sp. ArHS	1489488	1039	15	37	34	GTTTCAACCCACGCTCCCCGTTGCCGGGGAGCGATGC
